# Pan‐cancer analysis reveals molecular signatures for predicting matrix stiffness in solid tumors

**DOI:** 10.1002/ijc.70175

**Published:** 2025-09-24

**Authors:** Gongyu Tang, Xinyi Liu, Yuanxiang Li, Yunfei Ta, Minsu Cho, Hua Li, Xiaowei Wang

**Affiliations:** ^1^ Department of Pharmacology and Regenerative Medicine University of Illinois at Chicago Chicago Illinois USA; ^2^ Department of Mechanical Engineering and Materials Science Washington University in St. Louis St. Louis Missouri USA; ^3^ Department of Biomedical Engineering University of Illinois at Chicago Chicago Illinois USA; ^4^ Department of Radiation Oncology Washington University in St. Louis St. Louis Missouri USA; ^5^ Department of Bioengineering University of Illinois Urbana‐Champaign Urbana Illinois USA; ^6^ University of Illinois Cancer Center Chicago Illinois USA

**Keywords:** extracellular matrix, RNA‐seq, tumor matrix stiffness, tumor microenvironment, tumor rigidity

## Abstract

Tumor matrix stiffness plays a critical role in cancer progression, metastasis, and therapy resistance. Although traditional biophysical methods have shed light on the impact of matrix stiffness on tumor behavior, these techniques are confined to measuring the physical properties of the tumors. In this study, we leveraged RNA‐seq data to predict tumor matrix stiffness, aiming to reveal mechanical properties by molecular signatures across various cancer types. To this end, we systematically analyzed RNA‐seq data from tumors of varying stiffness levels to identify stiffness‐associated gene signatures. With these molecular signatures, we developed a computational model for predicting tumor matrix stiffness and further applied it to The Cancer Genome Atlas (TCGA) dataset. Our analysis revealed significant differences in the tumor microenvironment as well as immune response between soft and stiff tumor samples, suggesting that tumor rigidity impacts not only cellular behavior but also characteristics of the tumor microenvironment. These findings underscore the potential of RNA‐based stiffness models to enhance our comprehension of tumor mechanics and cancer biology, thereby facilitating the development of innovative targeted therapies.

## Graphical abstract

What's new?

Tumor matrix stiffness plays a critical role in cancer progression, metastasis, and therapy resistance. Here, the authors leveraged tumor RNA‐seq data to identify molecular signatures of mechanical properties across various cancer types and build a computational model for predicting tumor matrix stiffness. When applying their model to The Cancer Genome Atlas dataset, they found that tumor rigidity impacts not only cellular behavior but also characteristics of the tumor microenvironment. These findings underscore the potential of RNA‐based stiffness models to enhance the comprehension of tumor mechanics and cancer biology, thereby facilitating the development of innovative targeted therapies.

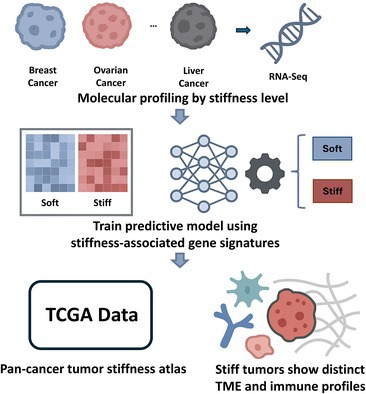

**Abbreviations**
AUC: area under the ROC curve
BLCA: bladder cancer
CAF: cancer‐associated fibroblast
COAD: colorectal adenocarcinoma
DEG: differentially expressed gene
ECM: extracellular matrix
EMT: epithelial‐mesenchymal transition
ESCA: esophageal carcinoma
GBM: glioblastoma multiforme
GEO: gene expression omnibus
GO: gene ontology
GSEA: gene set enrichment analysis
LGG: lower‐grade glioma
OV: ovarian cancer
PR: precision‐recall
READ: rectal adenocarcinoma
ROC: receiver operating characteristic
SKCM: skin cutaneous melanoma
STAD: stomach adenocarcinoma
TCGA: The Cancer Genome Atlas
THCA: thyroid carcinoma
TIDE: tumor immune dysfunction and exclusion
TMB: tumor mutation burden
TME: tumor microenvironment
TPM: transcripts per million
Treg: T regulatory cell

## 1 INTRODUCTION

Tumor matrix stiffness refers to the mechanical rigidity of a tumor, which is primarily influenced by the composition and structure of the extracellular matrix (ECM), including factors such as collagen cross‐linking and cellular contractility.^1^ Increased stiffness is linked to various oncogenic processes, affecting tumor initiation, progression, metastasis, and importantly, therapy resistance.^2^ Studying tumor rigidity is crucial as it not only contributes to our understanding of tumor biology but also opens up new avenues for therapeutic interventions that target the mechanical properties of the tumor microenvironment (TME).^3^

To study tumor matrix stiffness, researchers have employed advanced biophysical techniques such as atomic force microscopy and engineered hydrogels that mimic the mechanical characteristics of the ECM.^4^ For instance, microscopy combined with protein‐conjugated hydrogel substrates has been employed to show that ECM stiffness correlates with increased growth and migratory velocity in melanoma cells with high metastatic potential.^5^ Additionally, microscopy techniques have been used to demonstrate that matrix stiffness activates specific transcription factors in both cancer and stromal cells, regulating cancer progression in hepatocellular carcinoma.^6^ These findings highlight the critical role of ECM stiffness in cancer biology, revealing that biophysical methods can provide valuable insights into the mechanical forces driving tumor progression.

Beyond microscopy, researchers are increasingly employing next‐generation sequencing techniques, such as RNA‐seq, to investigate how tumor rigidity impacts functional pathways at the molecular level. For example, RNA‐seq has revealed previously unknown signatures, such as PLEC and TNS2, in the stiffness response in epidermal ovarian cancers, offering new insights into EOC metastasis in clinical settings.^7^ Additionally, RNA‐seq has shown that elevated ECM stiffness induces a malignant phenotype in breast cancer cells, with mRNA expression changes mirroring the transition from ductal carcinoma in situ to invasive ductal carcinoma, underscoring the role of stiffness in promoting cancer invasion.^8^ These findings suggest that RNA expression can be impacted by tumor matrix stiffness, supporting the feasibility of building stiffness prediction models based on transcriptomic data. However, to date, there are no systematic studies that attempt to predict the level of matrix stiffness using RNA‐seq data. This important knowledge gap underscores the need for further research that directly links transcriptomic data with matrix stiffness across various cancers.

In this study, we aim to leverage RNA‐seq data to predict tumor matrix stiffness by identifying gene markers associated with varying stiffness levels across multiple cancer types. Using RNA‐seq data from multiple cancer cell models, we identified general gene markers and developed a computational model to predict ECM stiffness. Applying this model to The Cancer Genome Atlas (TCGA) data,^9^ we established a pan‐cancer atlas describing tumor matrix stiffness. In this way, we observed notable differences in TME and immune response between soft and stiff tumor samples. These results underscore the promise of RNA‐based stiffness models in deepening our comprehension of tumor mechanics and their impact on cancer biology, opening avenues for novel targeted therapies.

## 2 METHODS

### 2.1 RNA‐seq data retrieval

**FIGURE 1:**
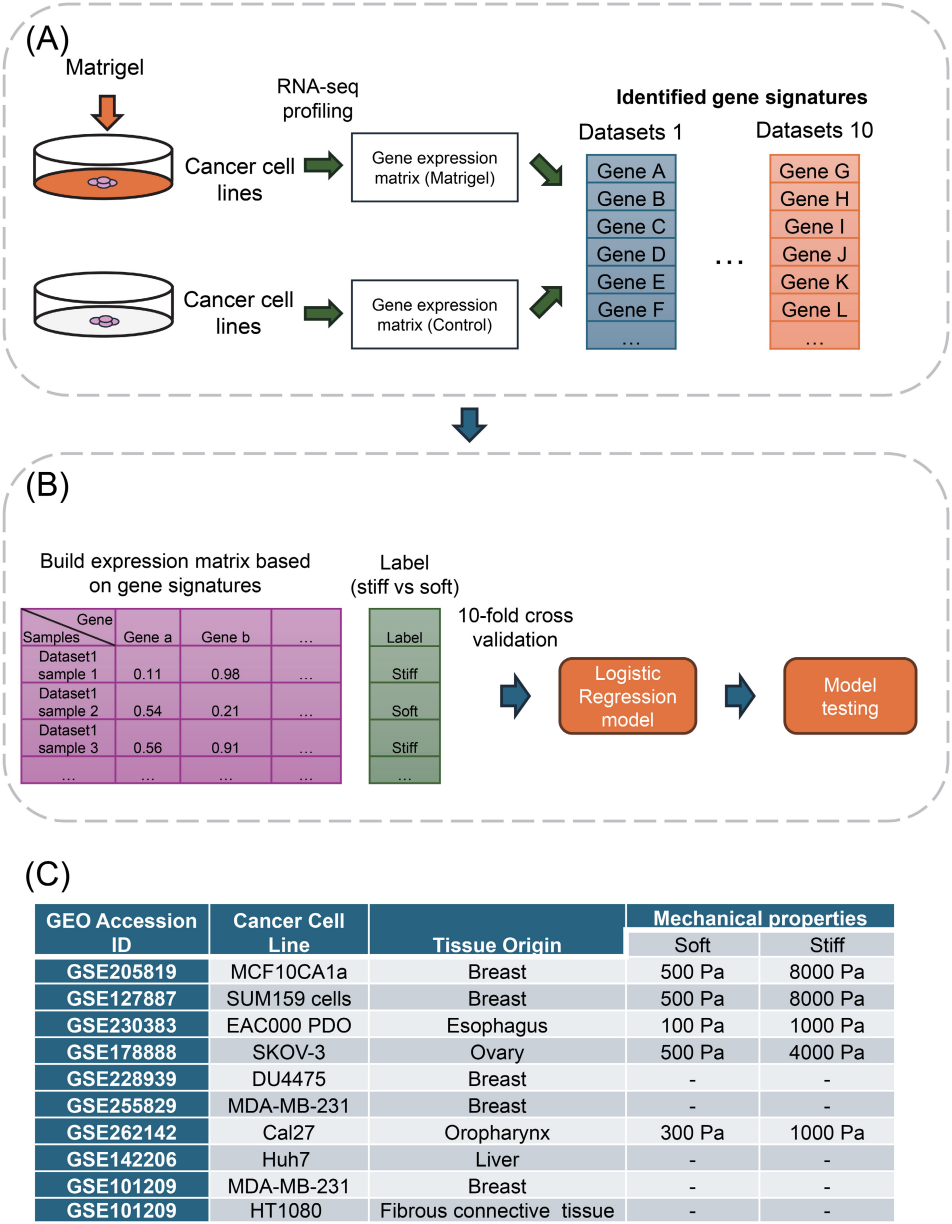
Workflow of the data analysis conducted in this study. (A) Workflow illustrating the process of identifying gene signatures associated with tumor matrix stiffness. (B) Workflow outlining the development of a computational model for predicting tumor matrix stiffness. (C) Summary of 10 GEO datasets included in model training, with the mechanical properties and tissue origins of the cancer cell lines presented.

We compiled a collection of RNA‐seq datasets from 10 independent studies that profiled tumor cells under various matrix stiffness conditions (Figure 1A). All datasets utilized engineered Matrigel to simulate different mechanical environments, as summarized in Figure 1C. These tumor cells originated from diverse tissues, including breast,^8^, ^10^, ^11^ ovary,^12^ esophagus,^13^ liver,^14^ oropharynx,^15^ and fibrous connective tissue.^16^ All these public datasets were retrieved from the Gene Expression Omnibus (GEO) database. Low concentrations of Matrigel, ranging from approximately 0.5–2.5 mg/mL, were used to mimic a normal tissue environment with stiffness values between 100 and 1000 Pa. High concentrations of Matrigel, ranging from around 6–12 mg/mL, were used to create a stiffer matrix environment with stiffness values ranging from 1000 to 8000 Pa. For example, MCF10CA1a cells (GSE205819) from breast tissue were cultured under soft (400 Pa) and stiff (5000 Pa) conditions, while SUM159 cells (GSE127887) from breast tissue were cultured under 500 Pa (soft) and 8000 Pa (stiff) conditions. Other cell lines, such as MDA‐MB‐231 (breast), Huh7 (liver), and HT1080 (fibrosarcoma), did not have specified mechanical stiffness values presented previously. However, similar to other public studies, differential concentrations of Matrigel were applied to these cell lines to simulate variations in tumor matrix stiffness.

### 2.2 RNA‐seq data analysis to identify marker genes for predicting matrix stiffness

RNA‐seq data were obtained from the GEO database using the SRA‐Toolkit. Raw RNA‐seq reads were aligned to the human reference genome GRch38.d1.vd1 (https://gdc.cancer.gov/about-data/gdc-data-processing/gdc-reference-files) using the STAR aligner.^17^ Gene expression profiles were determined with GENCODE version 35.0 (https://ftp.ebi.ac.uk/pub/databases/gencode/Gencode_human/release_35/gencode.v35.annotation.gtf.gz).^18^ Differential expression analysis was conducted with DESeq2^19^ to identify genes differentially expressed under soft vs. stiff matrix conditions. In this analysis, we adopted an adjusted *p*‐value <.05 and a log2 fold change >1 as significance thresholds. Genes identified as differentially expressed across multiple independent datasets were selected as common signature genes for matrix stiffness.

### 2.3 Computational model for predicting tumor matrix stiffness

We developed a logistic regression‐based model, StiffCalc, to predict tumor stiffness using the gene markers identified from 10 GEO RNA‐seq datasets as input features. Gene expression levels for all marker genes were normalized using the transcripts per million (TPM) method. To further reduce variability in gene expression across different datasets, we performed z‐score normalization for each gene within each dataset. This process transformed the gene expression values into standardized scores with a mean of 0 and a standard deviation of 1, ensuring that all gene expression levels were on a normalized scale before training the prediction model. Model training was performed using the R “glmnet” package,^20^ with normalized gene expression levels as input features and matrix stiffness as the outcome (i.e., classified as either soft or stiff). To improve robustness and optimize parameter estimation, the model was developed using the leave‐one‐out cross‐validation method. In this process, each of the 10 models was trained on nine datasets and tested on the remaining one in each iteration. Then, we averaged receiver operating characteristic (ROC) and the precision‐recall (PR) curve values to evaluate model performance. Feature importance was assessed by extracting the absolute values of the logistic regression coefficients, with higher absolute values indicating greater importance of the corresponding features.

### 2.4 TCGA pan‐cancer analysis

In this study, we analyzed 24 cancer types from the TCGA dataset, each containing over 100 tumor samples as well as at least 10 tumor‐adjacent samples as normal reference. We retrieved RNA‐seq (TPM reads) and mutation data from OncoDB^21^ to conduct the analysis. Using our computational model to predict tumor matrix stiffness, we categorized each tumor sample as either soft or stiff. We then compared the molecular signatures between these two groups to identify differences associated with tumor matrix stiffness. Matrisome and ECM genes were retrieved from MatrisomeDB.^22^ Based on MatrisomeDB annotations, six categories of matrisome genes were included in our study: glycoproteins, proteoglycans, collagens, secreted factors, ECM‐affiliated proteins, and ECM regulator genes.

### 2.5 Tumor microenvironment analysis

To investigate the impact of matrix stiffness on the TME, we utilized two computational methods: ESTIMATE and CIBERSORT. TPM‐normalized RNA‐seq reads were used as input for the ESTIMATE package to compute stromal and immune scores in tumor samples, providing a quantitative assessment of the tumor's non‐malignant cell content and overall purity. We compared three scores—stromal score, immune score, and ESTIMATE score—between the stiff and soft tumor groups. Similarly, CIBERSORT was used to deconvolve TPM‐normalized RNA‐seq data, estimating the relative abundance of 22 different cell types within the tumor samples. We compared the CIBERSORT‐derived cell score, fibroblast score, and endothelial score between the stiff and soft tumor groups. In this analysis, Student's *t*‐test was used to identify significant scores associated with matrix stiffness. Furthermore, log2‐fold change was calculated to highlight molecular signatures distinguishing stiff from soft tumors.

### 2.6 Gene set enrichment analysis

To investigate the biological pathways associated with matrix stiffness, we performed Gene Set Enrichment Analysis (GSEA)^23^ using the R “clusterProfiler” package.^24^ For each cancer type, we compared normalized gene expression levels between stiff and soft tumor samples. Genes with an average expression level below 5 were excluded from the GSEA analysis. We used a pre‐ranked list of genes, ordered by their differential expression (log2‐fold change) between stiff and soft tumor samples, to identify statistically significant key pathways associated with tumor rigidity. We conducted three separate GSEA analyses using the MSigDB, Reactome, and GO databases as references, respectively. Specifically, the MSigDB hallmark pathways, obtained from the “msigdbr” package, were included in our analysis. Significantly enriched pathways were categorized into three broad groups: immune‐related, metabolism‐related, and ECM‐related pathways. Similarly, pathways from the Reactome and GO databases were also extracted using the “msigdbr” package. For adaptive immune‐related pathway analysis, we specifically selected the Reactome pathway R‐HSA‐1280218.

To further investigate immune infiltration, we focused on a curated set of 17 immune cell marker genes,^25^ including those for B cells, macrophages, CD8+ T cells, T regulatory cells, and others. Similar to the pathway analysis, immune cell enrichment was calculated using the pre‐ranked list of genes. For both pathway analysis and immune cell enrichment analysis, significant features were identified by FDR‐adjusted *p*‐values. In our analysis, we only considered pathways/cell types with an absolute enrichment score >0.5 and an adjusted *p*‐value <.05.

### 2.7 Tumor immune dysfunction and exclusion analysis

The Tumor Immune Dysfunction and Exclusion (TIDE) analysis^26^ was conducted to assess the response to immune checkpoint inhibitors such as anti‐PD‐1 and anti‐CTLA4 therapies by predicting tumor immune evasion. First, TPM‐normalized RNA‐seq data from the OncoDB website were retrieved. Then, as a preprocessing step, these TPM values were log‐transformed and compared to those from matched adjacent normal samples. The fold change between tumor and normal samples was then log2 transformed to generate the input for TIDE. Response to immune checkpoint inhibitors was predicted with the TIDE score. To explore the influence of the TME on immune response, we compared the TIDE scores between tumors with a stiff matrix and those with a soft matrix within each cancer type using Student's *t*‐test. The resulting *p*‐values and log2‐fold changes were analyzed to identify significant differences in immune response across various matrix stiffness conditions.

## 3 RESULTS

### 3.1 Identifying common gene markers associated with tumor matrix stiffness

**FIGURE 2:**
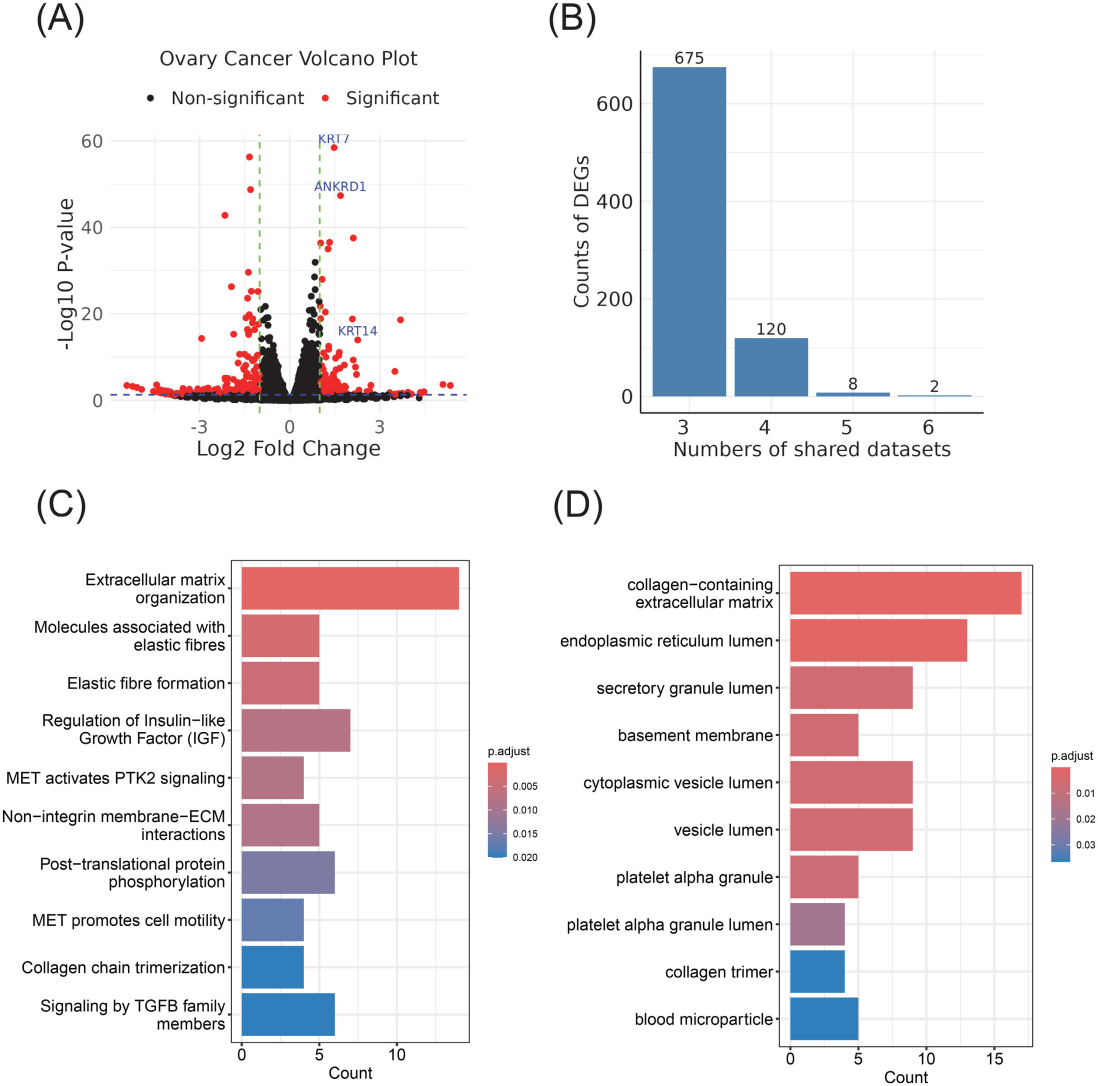
Summary of gene signatures associated with tumor matrix stiffness. (A) Volcano plot displaying DEGs in ovarian cancer, with significant genes highlighted in red. (B) Summary of commonly identified DEGs across the 10 datasets. (C) Pathway enrichment analysis of stiffness‐associated gene signatures using Reactome pathways. (D) Pathway enrichment analysis of stiffness‐associated gene signatures using gene ontology pathways.

To identify common gene signatures associated with matrix stiffness in cancer, we aggregated RNA‐seq datasets from 10 independent studies that analyzed tumor cells from various tissue origins (Figure 1C). These public studies focused on cancer cells with different mechanical properties by culturing them with engineered hydrogel (i.e., Matrigel) to simulate distinct mechanical environments. The “soft” culturing conditions ranged from 500 Pa to 1 KPa, while the “stiff” conditions ranged from 1 to 8 kPa. By comparing gene expression profiles in cancer cells under these two conditions, we identified differentially expressed genes (DEGs) in each dataset using DESeq2 (representative datasets shown in Figure 2A and Supplementary Figure S1A–D). Interestingly, this analysis revealed a subset of genes that were common DEGs across multiple datasets, such as KRT7 in both ovarian and breast cancers. Therefore, these shared DEGs are potential candidates for further investigation as general markers for predicting tumor matrix stiffness.

These DEGs associated with tumor matrix stiffness were shared at varying levels across all 10 datasets. Notably, 675 DEGs were identified in at least three datasets, and 120 were found in at least four datasets (Figure 2B). To further understand the functions of these stiffness‐related genes, we conducted pathway enrichment analysis on the 120 DEGs shared in at least four datasets. In this analysis, we performed pathway analysis on the 120 DEGs to capture broader trends and biological processes that are consistently enriched across multiple datasets. With the gene ontology (GO)^27^ and Reactome pathway databases^28^ as references, we discovered that these DEGs were significantly involved in biological processes and pathways crucial for the ECM, especially its structural components. Specifically, Reactome pathway analysis highlighted the enrichment of pathways involved in ECM organization, collagen formation, and membrane‐ECM interactions (Figure 2C). This suggests that ECM integrity and cell‐ECM interactions could contribute to tumor matrix stiffness. GO analysis further supported these results by identifying significant enrichment in pathways related to ECM components, tumor lumen, and cellular membrane components (Figure 2D). These structural elements are crucial for maintaining the mechanical strength of the ECM and facilitating effective cell‐matrix communication.^29^ Additionally, signaling pathways related to immune responses and metabolism were also enriched, suggesting that matrix stiffness is associated with not only the structural characteristics but also the immune response of the tumor cells.^30^ In summary, we found that common DEGs shared by multiple independent studies are associated with tumor ECM components and their organization, which further validated the utility of these DEGs as markers for predicting matrix stiffness and associated impact on the TME.

### 3.2 Developing a computational model for predicting tumor matrix stiffness

**FIGURE 3:**
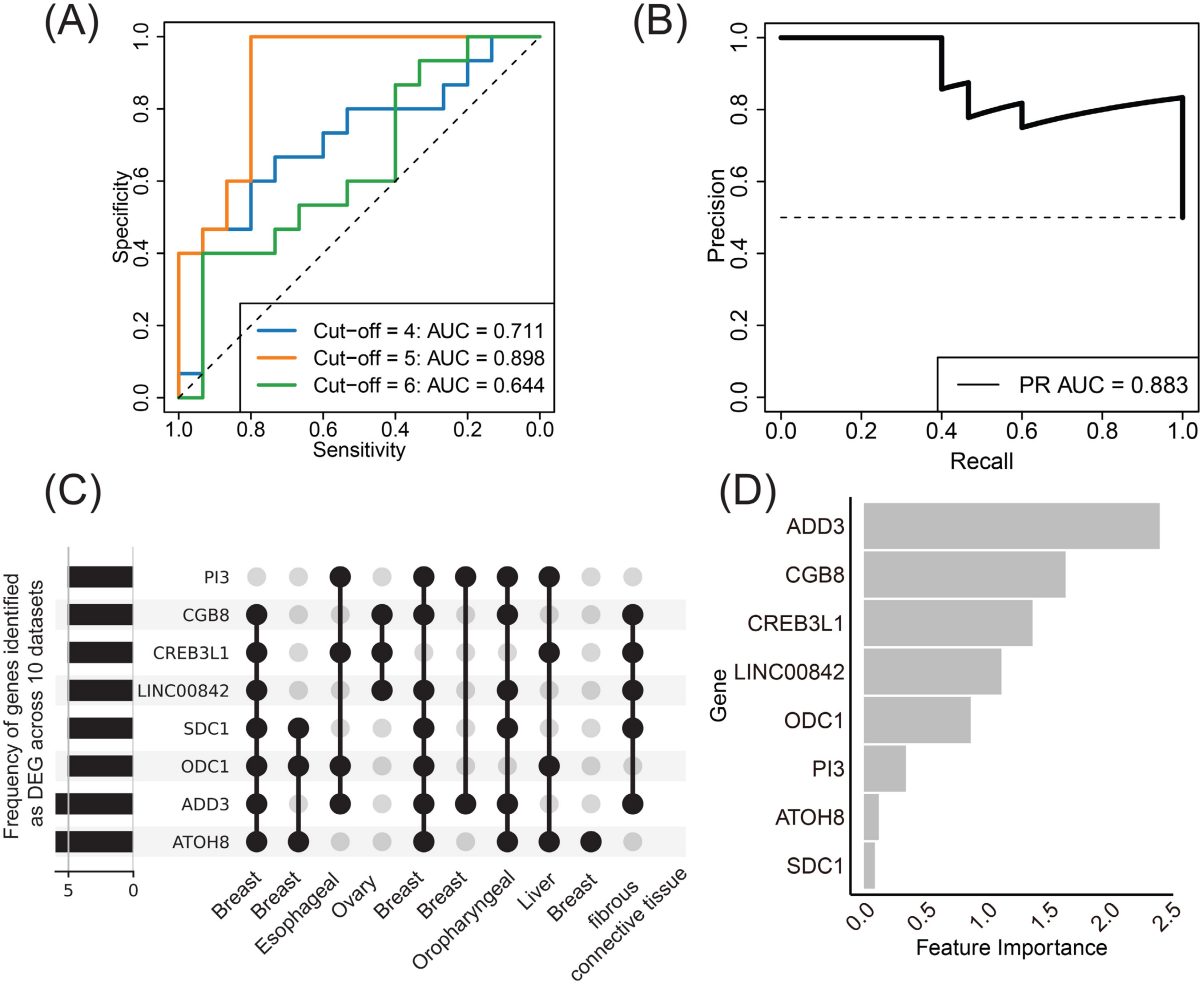
Evaluation of computational models for predicting tumor matrix stiffness. (A) ROC curves illustrating the performance of the computational models built with different sets of DEGs. The models were constructed using the DEGs identified in four to six out of the 10 datasets. (B) Precision‐Recall (PR) curve for evaluating the final model, StiffCalc. (C) Eight gene signatures consistently identified across the 10 datasets. (D) Feature importance analysis of the gene signatures for model development.

With the identified common DEGs associated with tumor matrix stiffness, we developed prediction models to differentiate between stiff tumors versus soft ones (Figure 1B). Three sets of common DEGs (shared across four, five, and six datasets, respectively) were used as input for model development. To this end, we employed a logistic regression model for its robust performance in binary classification. To optimize the model parameters and identify the most relevant common DEGs, we conducted leave‐one‐out cross‐validation, building 10 models by training on nine datasets and testing on a reserved dataset in each iteration. The average performance across all iterations was calculated and used for the final evaluation. ROC curves were employed to assess the performance of the models built with different DEG sets (Figure 3A). The model based on DEGs shared across five datasets had the best performance, resulting in an area under the ROC curve (AUC) of 0.893, compared to AUCs of 0.711 and 0.644 for the other two models. Consistently, the PR curve also demonstrated the best performance of this model, with an AUC of 0.883 (Figure 3B).

In summary, StiffCalc was modeled based on eight common stiffness‐associated genes: *ODC1*, *SDC1*, *LINC00842*, *CREB3L1*, *CGB8*, *PI3*, *ATOH8*, *ADD3* (Figure 3C). Feature importance analysis further revealed that ADD3 had the highest significance in the model, indicating its strongest association with matrix stiffness (Figure 3D). Traditionally, ADD3 is recognized for its role as a membrane cytoskeletal protein that strengthens the plasma membrane and supports various cellular physiological processes through signal transduction.^31^ Other significant genes identified in the model include *CGB8*, *CREB3L1*, *LINC00842*, *ODC1*, and *PI3*. *CGB8*, a glycoprotein hormone, is known for activating ECM‐related pathways that enhance tumor migration and invasion.^32^ *CREB3L1* is implicated in tumor growth and metastasis through the activation of ECM signaling pathways, remodeling the tumor microenvironment.^33^ *LINC00842* is associated with calcium ion binding, potentially leading to metabolic remodeling and alterations in the tumor microenvironment.^34^ *ODC1* promotes tumor cell proliferation and mobility via the AKT/GSK3β/β‐catenin pathway.^35^ *PI3*, a specific inhibitor of neutrophil elastase, binds to ECM proteins, trapping and neutralizing enzyme activity within the ECM.^36^ These genes are essential for maintaining ECM stiffness and driving tumor progression. Thus, by leveraging these genes to develop a predictive model, we could identify and categorize tumor samples based on varying levels of matrix stiffness.

To further demonstrate the stability and generalizability of the identified matrix stiffness markers, we included four additional independent datasets for further biomarker validation (Supplementary Table S1). These new datasets encompass diverse cancer types (including glioma, neuroblastoma, cervical, and breast cancers), ensuring a broader evaluation of our model's performance. These additional datasets demonstrate an average accuracy of 0.94, highlighting the robustness and generalizability of the StiffCalc model across various cancer types.

### 3.3 Variations in tumor complexity associated with matrix stiffness

**FIGURE 4:**
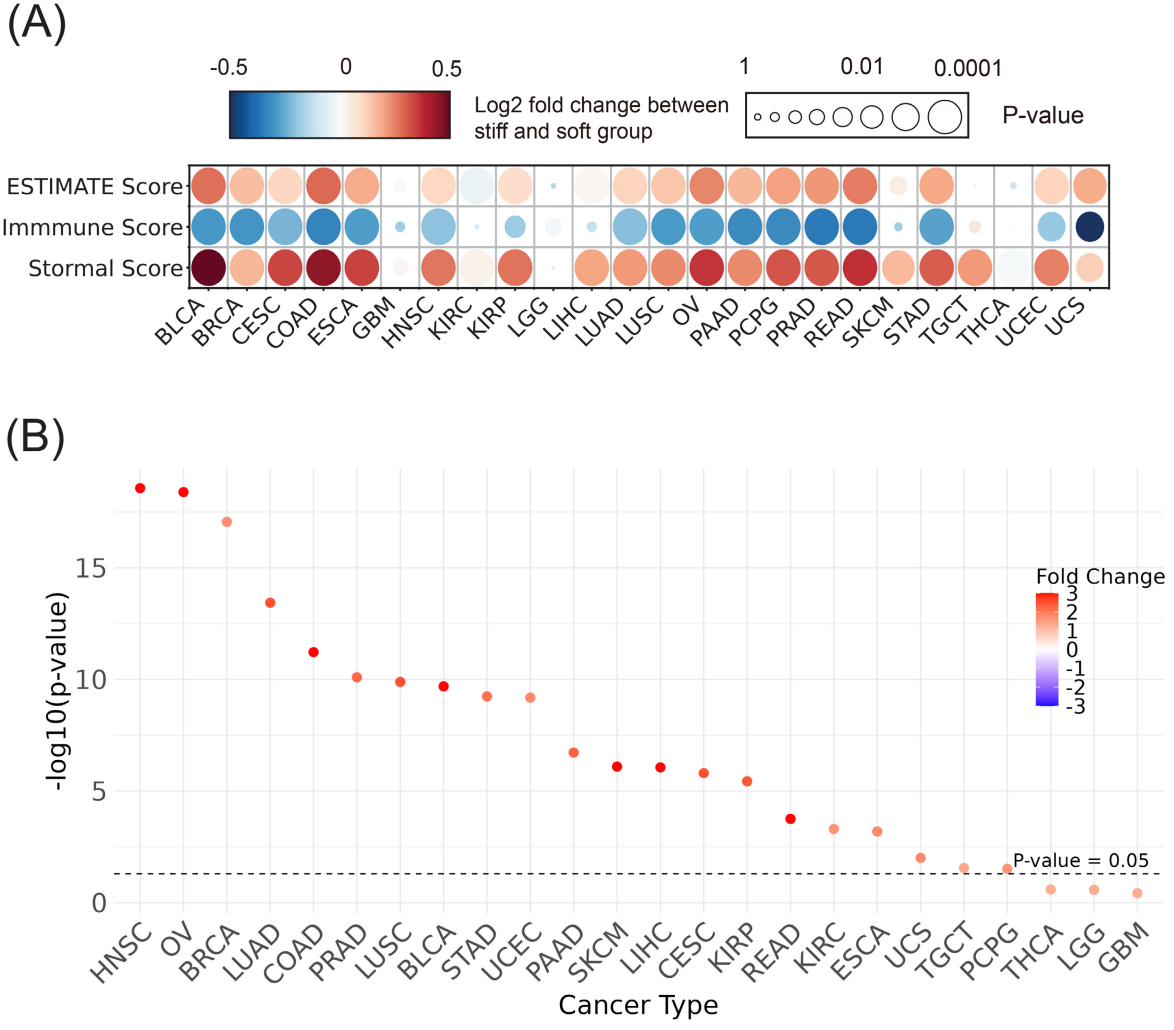
Pan‐cancer analysis of tumor complexity in relation to tumor matrix stiffness. (A) Variations in immune score, stromal score, and overall tumor complexity across different cancer types. The dot size indicates the *p*‐value from Student's *t*‐test comparing stiff and soft tumor groups, while the dot color represents log2‐fold change between these two groups. (B) Difference in cancer‐associated fibroblast scores across various cancer types between stiff and soft tumor groups. The dot color represents log2‐fold change between these two groups.

As described above, StiffCalc could robustly predict the matrix stiffness with cancer cell line models. As the biomarker genes included in the model were highly enriched in ECM‐related activities, we reasoned that the same model could also be applied to clinical tumor tissues for ECM stiffness prediction. To this end, we applied StiffCalc to the TCGA pan‐cancer dataset, analyzing 24 different cancer types to investigate the relationship between ECM stiffness and tumor complexity. The tumor samples were classified as either stiff or soft based on the model's predictions. We then employed the ESTIMATE package^37^ to assess tumor complexity by three different scoring schemes: ESTIMATE score, immune score, and stromal score. The ESTIMATE score is a composite score computed based on the immune and stromal scores, and it represents overall tumor complexity, with higher scores indicating more complex TME. In this way, we observed significant differences in tumor complexity between stiff and soft tumor groups across 21 of the 24 cancer types (Figure 4A). Interestingly, stiff tumors demonstrated higher ESTIMATE scores in 20 out of the 21 significant cancer types compared to soft tumors, indicating that stiff tumors have a more complex TME with a higher proportion of non‐tumor cells. In particular, stiff tumors showed higher stromal scores, indicating increased stromal cell infiltration, which is associated with a denser ECM and greater mechanical rigidity. Conversely, immune scores were generally lower for stiff tumors, suggesting reduced immune cell infiltration. This may indicate an immunosuppressive microenvironment often linked to a stiff tumor matrix. Additionally, for multivariate adjustments, we assessed multiple common clinical and molecular features (Supplementary Table S2), including stage, age, sex, race, and tumor mutation burden, against tumor complexity scores across 24 types of cancer included in our cohort. In univariate analysis, tumor stiffness, stage, and TMB were significantly positively correlated with tumor complexity. In multivariate analysis, these variables remained significantly correlated, confirming that tumor stiffness is independently associated with tumor complexity and tumor microenvironment changes.

Of the 21 significant cancer types revealed by ESTIMATE analysis, the most significant ones include bladder cancer (BLCA), colorectal adenocarcinoma (COAD), ovarian cancer (OV), and rectal adenocarcinoma (READ). However, in a few cancer types, including lower‐grade glioma (LGG), glioblastoma multiforme (GBM), and thyroid carcinoma (THCA), no significant difference in tumor complexity was associated with the stiffness scores, likely reflecting the unique biological and microenvironmental characteristics of these tumors. For example, the brain environment in GBM and LGG is known for its immune privilege, characterized by limited immune cell infiltration and distinct ECM components that differ significantly from those in other tissues.

To further investigate the differences in cellular composition between stiff and soft tumors, we employed CIBERSORT^38^ analysis to assess cancer‐associated fibroblasts (CAFs) and endothelial cells. The analysis revealed that stiff tumors consistently displayed higher scores for both CAFs and endothelial cells compared with soft tumors in 21 of the 24 cancer types (Figure 4B and Supplementary Figure S2). This finding suggests that CAFs, known for their role in actively remodeling the ECM and increasing tissue rigidity, may play a crucial role in establishing and maintaining the matrix stiffness. Additionally, the elevated presence of endothelial cells in stiff tumors underscores the significance of vascular architecture in shaping the mechanical properties of the tumor. The combined effects of these cells highlight the complex interplay within the TME, where the mechanical properties of the tumor are closely linked to its cellular composition as well as the dynamic interactions between stromal and immune components. Thus, understanding these TME differences is crucial for developing novel therapeutic strategies that target the biomechanical properties of the tumors, potentially leading to more effective cancer treatments.

### 3.4 Pathway analysis of the functional effects of tumor matrix stiffness

**FIGURE 5:**
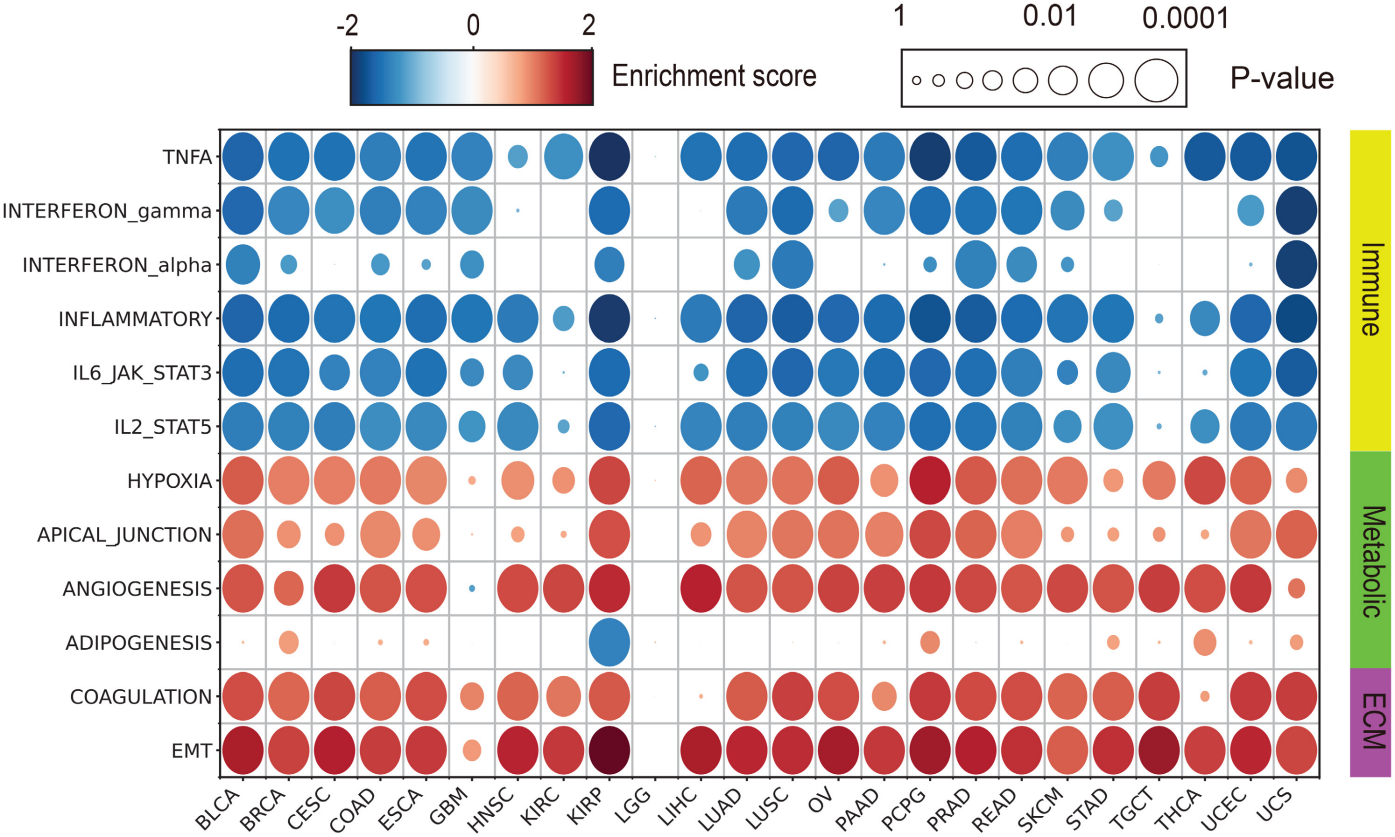
Pan‐cancer GSEA analysis of stiff versus soft tumors. Top significant pathways from MSigDB are presented. The dot size reflects the adjusted *p*‐value (FDR) from GSEA comparing stiff and soft tumor groups, while the dot color represents the log2‐fold change between these two groups.

To investigate the biological processes affected by tumor rigidity, we performed a pathway enrichment analysis using the MSigDB hallmark pathways as a reference.^23^ Stiff tumors demonstrated significant enrichment in apical junction and epithelial‐mesenchymal transition (EMT) pathways (Figure 5), suggesting that increased tumor rigidity is associated with alterations in cell adhesion and migration. Additionally, stiff tumors exhibited significant enrichment in metabolic pathways associated with hypoxia and angiogenesis, indicating that alterations in the mechanical properties of the tumor samples may impact vascular dynamics and oxygenation. Notably, immune‐related pathway activities, including TNFα signaling, interferon pathways, and IL6‐JAK‐STAT3 signaling, were reduced in stiff tumors, suggesting a diminished immune response potentially from altered structural characteristics of the tumor. These findings are consistent with our ESTIMATE analysis results, highlighting the potential role of tumor stiffness in shaping the TME and impacting key biological processes.

Given the general reduction in immune‐related activities in stiff tumors, we further focused specifically on adaptive immunity, a crucial component of the body's defense against cancer. Adaptive immunity, characterized by its antigen‐specific response, is essential for tumor recognition, immune evasion, and the development of personalized immunotherapies. To investigate the adaptive immune‐related response, we conducted a pathway analysis using the Reactome database. We observed a significant reduction in activity from certain adaptive immune pathways such as TCR signaling, PD‐L1, immunoregulatory interactions, and cytokine signaling (Supplementary Figure S3). The reduced activity of these immune pathways in stiff tumors suggests that increased tumor rigidity may contribute to an impaired adaptive immune response. This could potentially facilitate immune evasion by the tumor, thus presenting challenges for effective immunotherapy.^39^ On the other hand, we did not observe significant changes in B cell activation or antigen presentation (as represented by BCR signaling and MHC class I pathways, respectively).

Overall, these findings demonstrate that tumor stiffness is intricately connected to key biological processes such as EMT, angiogenesis, and immune response. Increased tumor rigidity could potentially promote tumor cell migration, invasion, and the formation of new blood vessels. Meanwhile, by modulating immune activities, a stiff ECM may facilitate immune evasion and resistance to immune surveillance. These findings emphasize the crucial role of tumor rigidity in disease progression, underscoring its significance in developing effective therapeutic strategies.

### 3.5 Immune infiltration patterns in relation to tumor matrix stiffness

**FIGURE 6:**
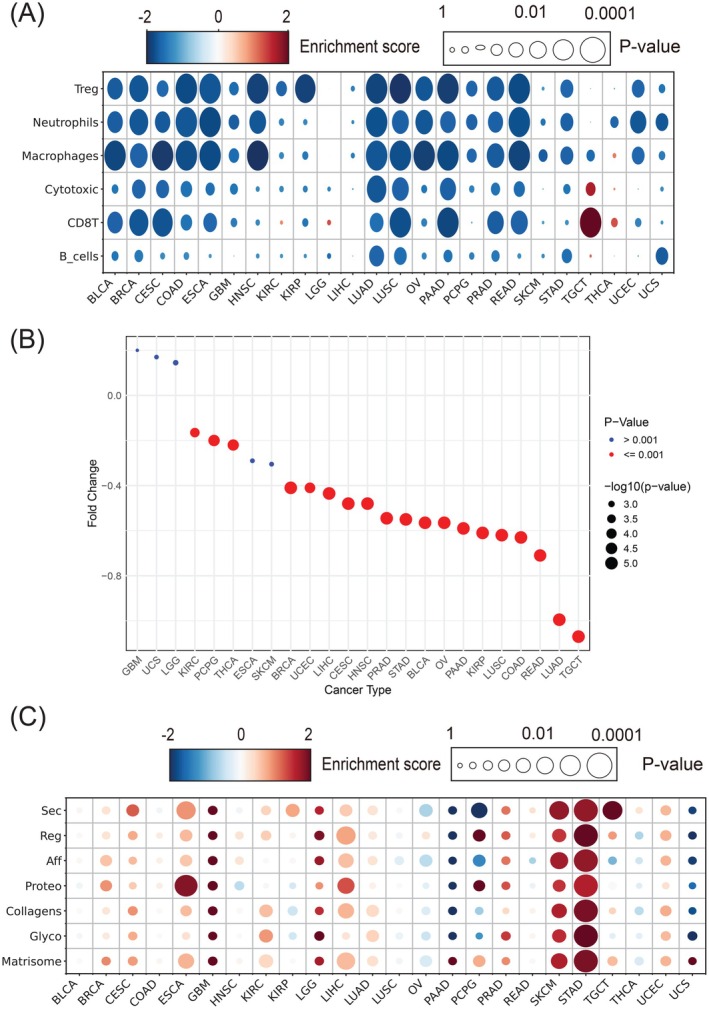
Pan‐cancer immune infiltration and matrix mutation analyses of stiff versus soft tumors. (A) GSEA analysis based on the expression of immune cell marker genes. (B) Fold change of TIDE prediction scores between stiff and soft tumors. (C) Pan‐cancer analysis of matrisome mutations in relation to tumor matrix stiffness. Matrisome mutations in most cancers were positively correlated with increased matrix stiffness.

As the immune activities were significantly associated with tumor rigidity, we further determined how changes in the mechanical properties of tumor samples affect their immune infiltration patterns. To this end, we selected 17 immune cell types along with their marker genes and compared the gene set enrichment scores between stiff and soft tumors. The most significantly altered immune cells, which were depleted in stiff tumors, are presented in Figure 6A. Specifically, key immune cells such as T regulatory cells (Tregs), neutrophils, macrophages, cytotoxic/CD8+ T cells, and B cells exhibited markedly lower enrichment scores in stiff tumors across multiple cancer types. Notably, the depletion of T cells, particularly cytotoxic / CD8+ T cells, was more pronounced in stiff tumors compared to B cells. This suggests a selective suppression or exclusion of specific T cell subpopulations within the stiff tumor microenvironment. The observed depletion of T cells in stiff tumors correlated with diminished TCR signaling activity (Supplementary Figure S3), reinforcing the link between tumor rigidity and impaired immune functions. Thus, these findings support the notion that matrix stiffness may interfere with adaptive immune responses, leading to an immunosuppressive microenvironment that hinders T cell‐mediated tumor eradication.

Given the significant association between tumor rigidity and immune activities, we further employed TIDE^26^ to predict therapeutic immune response for tumors with varying levels of rigidity. In this analysis, a lower TIDE score predicted a higher likelihood of immune evasion, leading to reduced response to treatment. Consistent with the observed immune cell depletion, the TIDE analysis results suggested that most stiff tumors were more likely to be resistant to immunotherapies (Figure 6B). This finding highlights the need to account for tumor rigidity when designing and enrolling patients for immunotherapies, as it can significantly affect immune cell infiltration into the TME, potentially reducing treatment efficacy.

We further conducted correlative analyses using TCGA pan‐cancer data to examine the potential relationship between tumor stiffness and common clinical features. Specifically, we focused on tumor stage, which is reliably recorded at diagnosis in 11 types of cancer (Supplementary Table S3). We stratified the patients into two groups: those with tumor stages I to III and those with tumor stage IV. Our findings indicated that stiff tumors were significantly associated with more advanced tumor stages in five of the 11 cancer types, suggesting that stiffer tumors tend to have a more advanced stage.

### 3.6 Matrisome mutations associated with tumor matrix stiffness

Tumor mutations are pivotal in both the initiation and progression of tumors. In particular, mutations in matrisome genes, which encode ECM proteins and related factors, can alter the mechanical properties of the tumor. To investigate this, we performed a comparative analysis of all gene mutations versus matrisome‐specific mutations across different cancer types. Interestingly, matrisome genes exhibited a significantly higher mutation burden compared to the overall gene mutation burden in the tumors (3.08 vs. 4.35 for matrisome and total genes, respectively). This finding prompted us to explore the correlation between specific types of matrisome mutations and matrix stiffness (Figure 6C). Our analysis revealed that most matrisome mutations were positively correlated with increased matrix stiffness. Tumors with a higher burden of matrisome mutations tended to exhibit greater stiffness, a correlation that was particularly pronounced in certain types of cancers, including skin cutaneous melanoma (SKCM), esophageal carcinoma (ESCA), and stomach adenocarcinoma (STAD). These mutations may directly impact the physical properties of the ECM, as suggested by previous studies. Additionally, we included Cell Cycle as a reference group. In contrast to subcategories of matrix genes, cell cycle genes are not related to tumor matrix stiffness except in one tumor type (TGCT).

We also identified individual genes in the matrisome that may directly influence matrix stiffness (Supplementary Figure S4). Notably, mutations in collagen genes and matrix metalloproteinases (MMPs) were more frequent in stiff tumors, particularly in STAD.^40^, ^41^ These mutations could potentially contribute to abnormal collagen cross‐linking, leading to increased ECM rigidity. Additionally, mutations in laminin and serpin,^42^ which are key components of the basement membrane and interstitial matrix, were highly enriched in STAD and SKCM. These mutations may alter cell‐ECM interactions, contributing to the mechanical resistance observed in stiff tumors. Thus, our analysis highlights the pivotal role of matrisome mutations in shaping matrix stiffness by modifying the ECM structure and functions.

## 4 DISCUSSION

In our study, we developed a model to predict tumor matrix stiffness based on RNA‐seq data. Traditionally, tumor matrix stiffness has been measured using biophysical techniques such as atomic force microscopy or magnetic resonance elastography. While effective, these methods are limited by their technical complexity, cost, and the need for specialized equipment. Our approach, by leveraging RNA‐seq data, offers a promising alternative that is both scalable and accessible. Predicting tumor matrix stiffness from gene expression profiles is particularly valuable for several reasons. First, RNA‐seq data are widely available, especially from large‐scale cancer genomics projects such as TCGA. The availability of large‐scale RNA‐seq data makes it feasible to integrate matrix stiffness with other tumor characteristics for comprehensive analysis of tumor behaviors. In particular, this integration enhances our understanding of how mechanical properties influence tumor behaviors at the molecular level. Second, identifying stiffness‐associated gene signatures provides critical insights into the molecular pathways underlying tumor rigidity. These pathways could be crucial for tumor progression, invasion, and resistance. Understanding these molecular mechanisms could thus help predict which tumors are more likely to exhibit increased stiffness, leading to more aggressive behaviors. Furthermore, at the gene level, we identified several new biomarkers related to tumor matrix stiffness. Of note, CGB8, a glycoprotein hormone β‐subunit primarily associated with pregnancy, was consistently differentially expressed across multiple datasets. While previous reports have linked CGB8 to ECM‐related pathways in prostate cancer, our findings suggest its broader involvement in many cancers, particularly in activating ECM‐related pathways that alter tumor stiffness. This novel observation extends the known biological role of CGB8 and highlights its potential significance in tumor stiffness across multiple cancer types.

By applying our model to TCGA data, we demonstrated significant differences in the TME and immune response between stiff and soft tumors. Stiff tumors were generally associated with a more complex and immunosuppressive microenvironment, characterized by higher stromal cell infiltration and lower immune cell presence. This finding suggests that tumor matrix stiffness not only impacts the physical properties of the tumor but also influences its biological behaviors, particularly its interaction with the immune system. Similar immune alterations due to tumor matrix stiffness have been previously reported in experimental studies.^43^ The mechanisms behind these observations are likely multifaceted. Tumor matrix stiffness could result in a denser ECM, which acts as a physical barrier to immune cell infiltration.^44^ Additionally, altered mechanical properties may activate signaling pathways that promote an immunosuppressive environment, further inhibiting the recruitment and activation of the immune cells.^45^ Our findings are further supported by experimental evidence from the discovery datasets, which consistently highlight key genes and pathways involved in ECM organization, including collagen formation and ECM remodeling. Furthermore, we observed heterogeneity across various cancer types, which reflects the differences in tumor biology and immune responses among them. Despite this variability, the trend remained consistent in 21 out of 24 cancer types, where stiffer tumors were linked to poorer predicted immunotherapy outcomes. This consistent pattern across the vast majority of cancer types strengthens our conclusion that stiffer tumors generally have worse immunotherapy outcomes.

Understanding the role of tumor matrix stiffness in shaping the immune microenvironment not only sheds light on tumor biology but also suggests potential implications for therapeutic strategies. For example, tumor rigidity could serve as a biomarker for predicting immunotherapy outcomes. Currently, tumor mutation burden (TMB) is widely used as a predictor for immunotherapy success^46^; similarly, our analysis indicates that stiff tumors tend to have worse immunotherapy outcomes. We propose that incorporating tumor matrix stiffness as an additional biomarker, alongside TMB, could improve patient stratification for immunotherapies and provide a more nuanced understanding of which tumors are most likely to benefit from these treatments.

Building on these findings, novel therapeutic strategies could be developed to target ECM components that contribute to stiffness, such as inhibiting collagen crosslinking to reduce tumor rigidity and improve immune cell infiltration.^47^ Furthermore, combining therapies that modulate tumor rigidity with immunotherapies, such as immune checkpoint inhibitors, could enhance the efficacy of these treatments by creating a more favorable immune microenvironment. For instance, previous studies have demonstrated that targeting ECM stiffness can improve the effectiveness of immune checkpoint inhibitors and adoptive cell therapies.^48^ By addressing both the mechanical and immune aspects of the tumor microenvironment, we could potentially overcome the resistance commonly observed in stiff tumors, leading to improved patient outcomes.

Clinically, the tumor stiffness model offers a promising tool for patient stratification, prediction of tumor aggressiveness, and guidance of personalized therapy. For example, tumors exhibiting high stiffness and low immune infiltration may be less responsive to immunotherapy alone, suggesting a potential benefit from combining ECM‐targeting agents with immune checkpoint inhibitors. In contrast, tumors with low stiffness and an inflamed immune microenvironment may be more amenable to immunotherapy monotherapy. By integrating stiffness scores with other molecular predictors, such as TMB, PD‐L1 expression, and cytokine profiles, clinicians could achieve improved risk assessment and treatment planning. Additionally, the model may be used to monitor therapy‐induced changes in tumor rigidity, offering dynamic insights into treatment efficacy.

While our current study focused on tumor matrix stiffness as a key determinant of aggressiveness, we recognize that a multimodal biomarker approach, by combining stiffness with immune and genomic features, could significantly enhance predictive performance. Another limitation of our study is that the tumor stiffness model was trained exclusively on cells of solid tumor origin. As such, its performance may not generalize to soft tissue tumors or hematologic malignancies, which differ significantly in biomechanical properties and tissue architecture. Future work will be necessary to evaluate the model's applicability across a wider range of tumor types.

## AUTHOR CONTRIBUTIONS

**Gongyu Tang:** Writing – review and editing; methodology; software; conceptualization; investigation; writing – original draft; data curation; formal analysis; project administration; supervision; resources; validation; visualization. **Xinyi Liu:** Conceptualization; writing – review and editing; validation; methodology; resources. **Yuanxiang Li:** Conceptualization; writing – review and editing; resources; data curation; investigation. **Yunfei Ta:** Methodology; resources; writing – review and editing; investigation. **Minsu Cho:** Investigation; writing – review and editing; resources; data curation. **Hua Li:** Conceptualization; funding acquisition; writing – review and editing; resources; supervision; project administration. **Xiaowei Wang:** Project administration; resources; supervision; data curation; writing – review and editing; writing – original draft; funding acquisition; investigation; conceptualization; methodology.

## FUNDING INFORMATION

This research was supported by the National Institutes of Health (R01DE026471, R01CA233873, R56DE033344, R01CA287778, and R35GM141535).

## CONFLICT OF INTEREST STATEMENT

The authors declare no conflict of interest.

## Supporting information


**Supplementary Table S1.** Performance of StiffCalc on independent validation datasets.
**Supplementary Table S2.** Multivariate analysis of tumor complexity (Estimate score).
**Supplementary Table S3.** Correlation between tumor stage and tumor matrix stiffness.
**Supplementary Figure S1.** Volcano plots displaying DEGs in various cancer types.
**Supplementary Figure S2.** Difference in endothelial cell scores across cancer types.
**Supplementary Figure S3.** Pan‐cancer GSEA analysis comparing stiff and soft tumors for.
**Supplementary Figure S4.** Pan‐cancer analysis of matrisome gene mutations associated.

## Data Availability

The source code and installation instructions for StiffCalc are available through GitHub (https://github.com/wang-lab/StiffCalc). Further information is available from the corresponding author upon request.
