# Supplementary material for: Pan‐cancer analysis reveals molecular signatures for predicting matrix stiffness in solid tumors
Source: Int J Cancer. 2025 Sep 24;158(4):1093–105. doi: 10.1002/ijc.70175 (PMC12712361; doi:10.1002/ijc.70175)
Supplement: Supplementary file 1 — Supplementary Table S1. Performance of StiffCalc on independent validation datasets. Supplementary Table S2. Multivariate analysis of tumor complexity (Estimate score). Supplementary Table S3. Correlation between tumor stage and tumor matrix stiffness. Supplementary Figure S1. Volcano plots displaying DEGs in various cancer types. Supplementary Figure S2. Difference in endothelial cell scores across cancer types. Supplementary Figure S3. Pan‐cancer GSEA analysis comparing stiff and soft tumors for. Supplementary Figure S4. Pan‐cancer analysis of matrisome gene mutations associated. [file IJC-158-1093-s001.pdf]

Pan-Cancer Analysis Reveals Molecular Signatures for Predicting Matrix Stiffness in Solid Tumors

Gongyu Tang, Xinyi Liu, Yuanxiang Li, Yunfei Ta, Minsu Cho, Hua Li, and Xiaowei Wang

Table of Content

- Supplementary Table S1. Performance of StiffCalc on independent validation datasets.
- Supplementary Table S2. Multivariate analysis of tumor complexity (Estimate score) across 24 types of cancer.
- Supplementary Table S3. Correlation between tumor stage and tumor matrix stiffness.
- Supplementary Fig. S1. Volcano plots displaying DEGs in various cancer types.
- Supplementary Fig. S2. Difference in endothelial cell scores across cancer types between stiff and soft tumor groups.
- Supplementary Fig. S3. Pan-cancer GSEA analysis comparing stiff and soft tumors for adaptive immune-related pathways from the Reactome database.
- Supplementary Fig. S4. Pan-cancer analysis of matrisome gene mutations associated with tumor matrix stiffness.

Supplementary Table S1. Performance of StiffCalc on independent validation datasets.

GEO Dataset	Cell line	Cancer cell type	Accuracy	Included DEGs
GSE182606	MDA-MB-231	Breast	1.00	5
GSE182606	U251G	Glioma	0.92	4
GSE246550	SH-SY5Y	Neuroblastoma	0.83	3
GSE83366	Hela	Cervix	1.00	4

Supplementary Table S2. Multivariate analysis of tumor complexity (Estimate score) across 24 types of cancer.

	Univariate		Multivariate	
	Fold change (log2)	P value	Fold change (log2)	P value
**Tumor stiffness score**	0.40	9.8E-17	0.35	5.1E-09
**Age**	-0.19	3.3E-01	-0.12	5.7E-01
**Stage**	0.33	2.6E-07	0.26	6.3E-05
**Sex**	-0.07	4.6E-01	-0.07	8.9E-01
**Race**	-0.04	6.7E-01	-0.03	9.8E-01
**Tumor mutation burden**	0.25	5.4E-03	0.20	1.1E-02

Supplementary Table S3. Correlation between tumor stage and tumor matrix stiffness.

Cancer type	Fold change (log2)	p-value
BLCA	2.93	1.60E-03
KIRC	0.91	5.80E-05
LGG	0.91	8.60E-04
STAD	1.46	8.20E-04
UCEC	0.78	1.80E-02

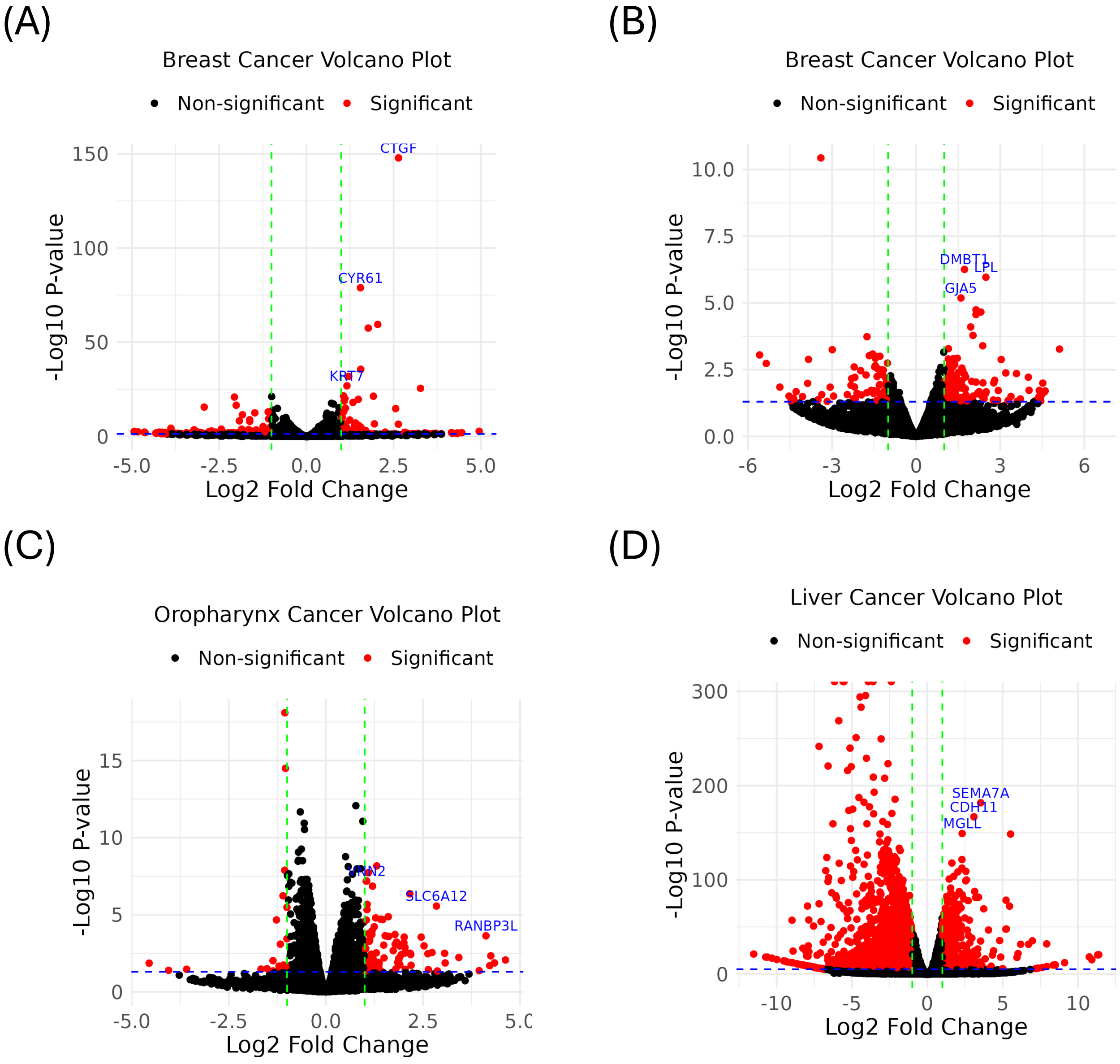

Supplementary Fig. S1. Volcano plots displaying DEGs in various cancer types, with significant genes highlighted in red. Four cancer types are presented, including breast (A), breast (B), oropharynx (C), and liver (D).

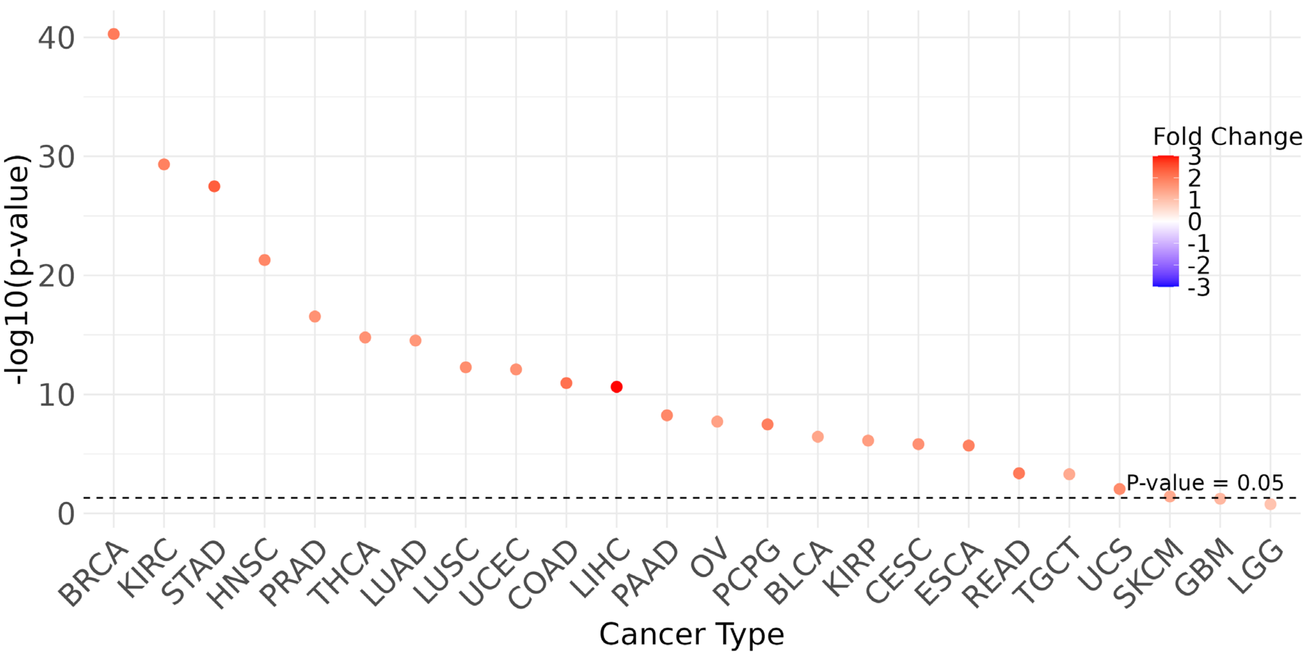

Supplementary Fig. S2. Difference in endothelial cell scores across cancer types between stiff and soft tumor groups. The dot color represents log2-fold change between these two groups.

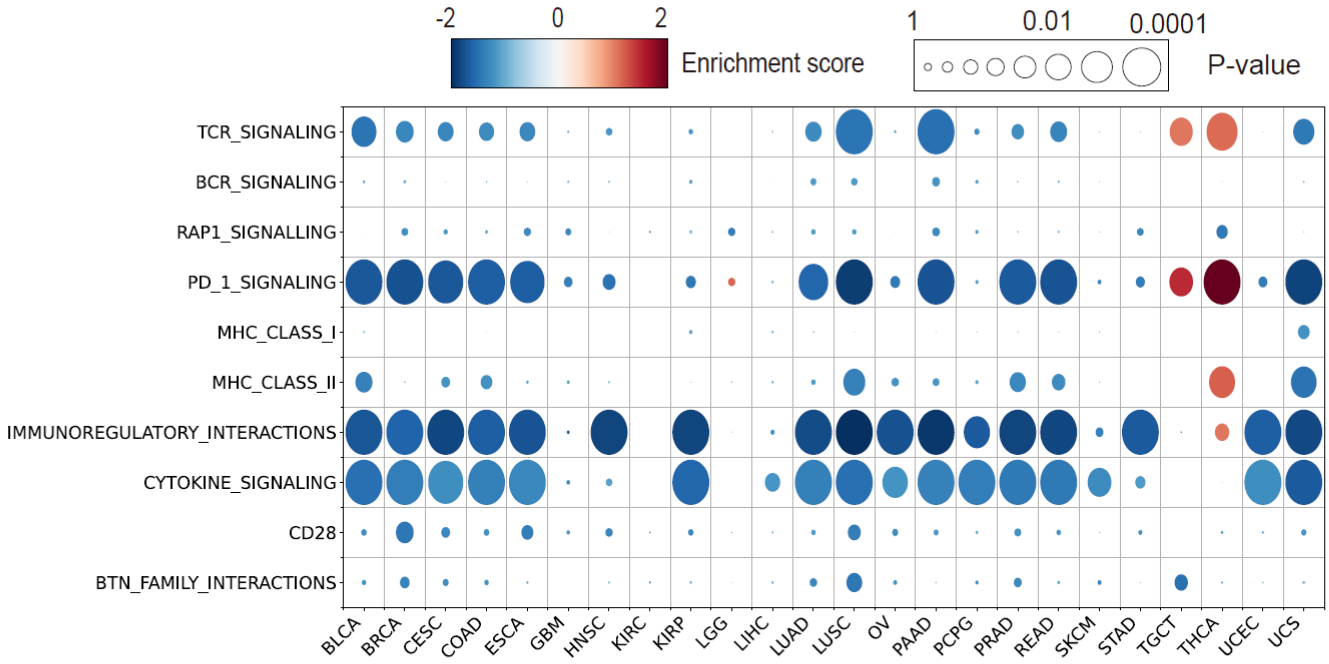

Supplementary Fig. S3. Pan-cancer GSEA analysis comparing stiff and soft tumors for adaptive immune-related pathways from the Reactome database. The dot size corresponds to the p-value from Student's t-test between the two groups, while the dot color indicates the log2-fold change between stiff and soft tumors.

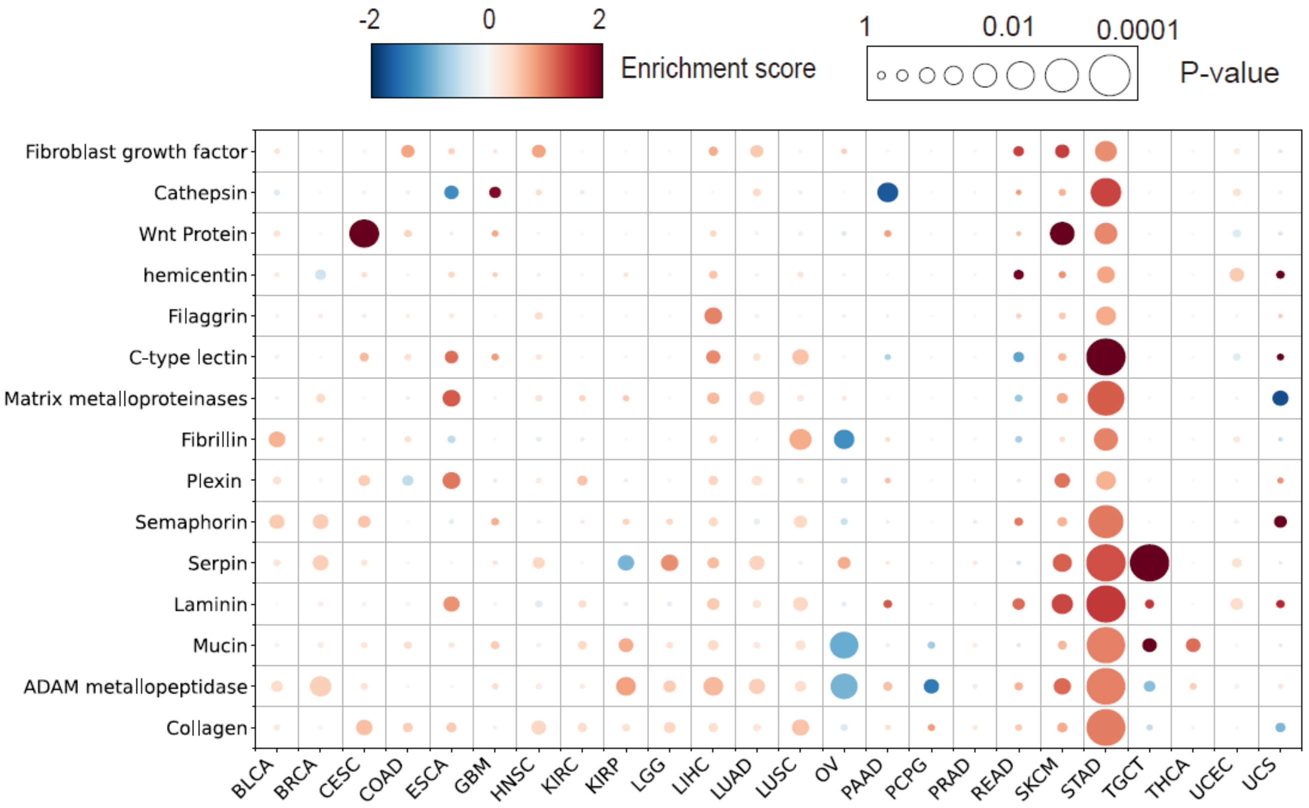

Supplementary Fig. S4. Pan-cancer analysis of matrisome gene mutations associated with tumor matrix stiffness. The dot size reflects the p-value from Student's t-test comparing stiff and soft tumor groups, while the dot color represents the log2-fold change between these two groups.
